# Supplementary material for: Metabolomics and machine learning technique revealed that germination enhances the multi-nutritional properties of pigmented rice
Source: Commun Biol. 2023 Oct 2;6:1000. doi: 10.1038/s42003-023-05379-9 (PMC10545681; doi:10.1038/s42003-023-05379-9)
Supplement: Supplementary file 2 — Supplementary Information [file 42003_2023_5379_MOESM2_ESM.pdf]

SUPPLEMENTARY FIGURES

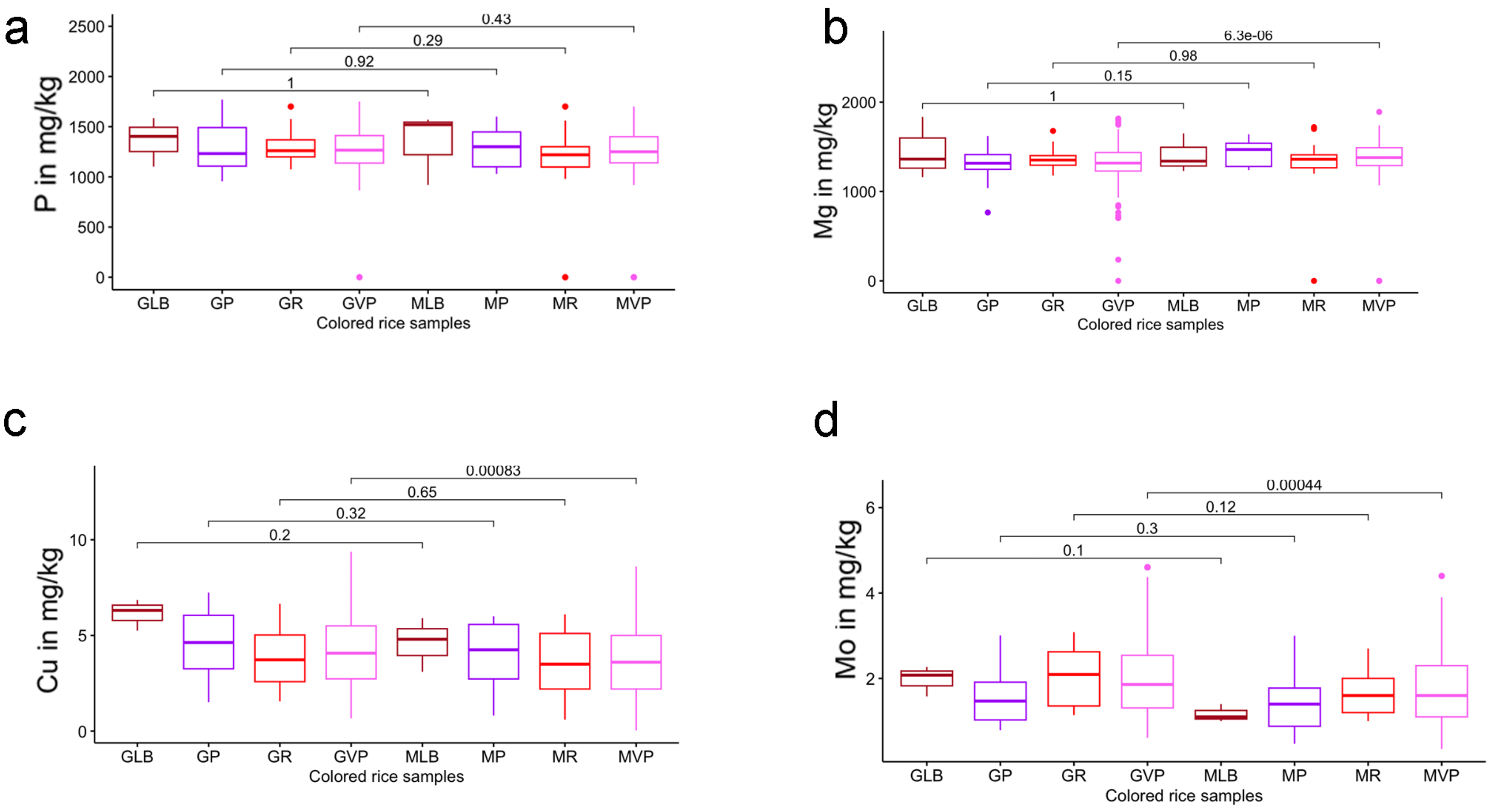

Supplementary Figure 1. No significant effects to other minerals in some rice types (in mg/kg): (a) phosphorus (P), (b) magnesium (Mg), (c) copper (Cu), (d) molybdenum (Mo). In the boxplot, the solid middle line depicts the median, while the lower and upper whiskers signify the 25th and 75th percentiles, respectively. (GLB = Germinated Light brown, GP = Germinated Purple, GR = Germinated Red, GVP = Germinated Variable Purple, MLB = Matured (non-germinated) Light brown, MP = non-germinated Purple, MR = non-germinated Red, MVP = non-germinated variable purple)

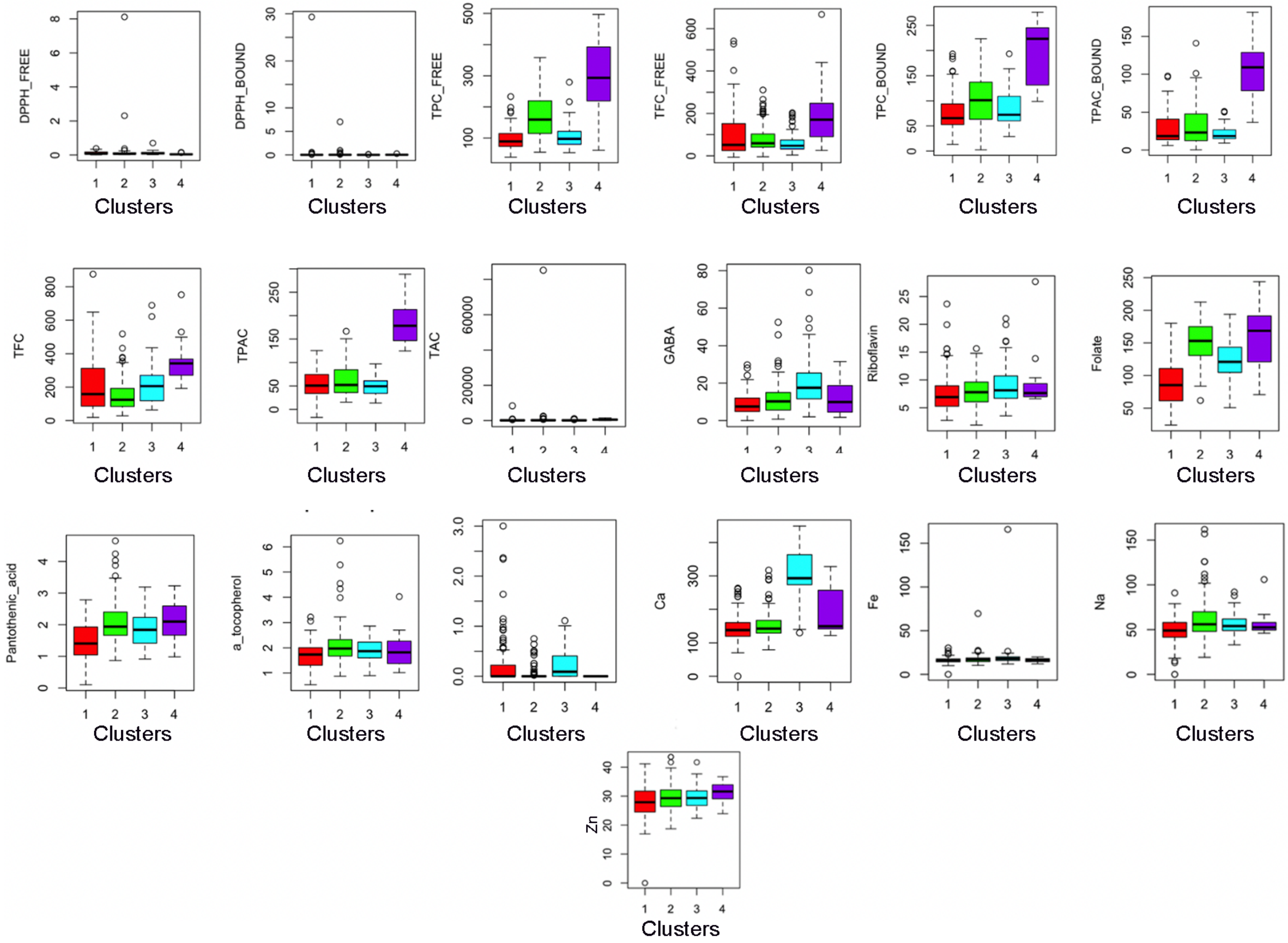

Supplementary Figure 2. Boxplots of the traits used in models and their levels in four clusters. DPPH free, DPPH bound, TPC free, TFC free, TPC bound, TPAC bound, TFC, TPAC, TAC, GABA, Riboflavin, Folate, Pantothenic acid, alpha tocopherol, biotin, Ca, Fe, Na, Zn. In the boxplot, the solid middle line depicts the median, while the lower and upper whiskers signify the 25th and 75th percentiles, respectively.

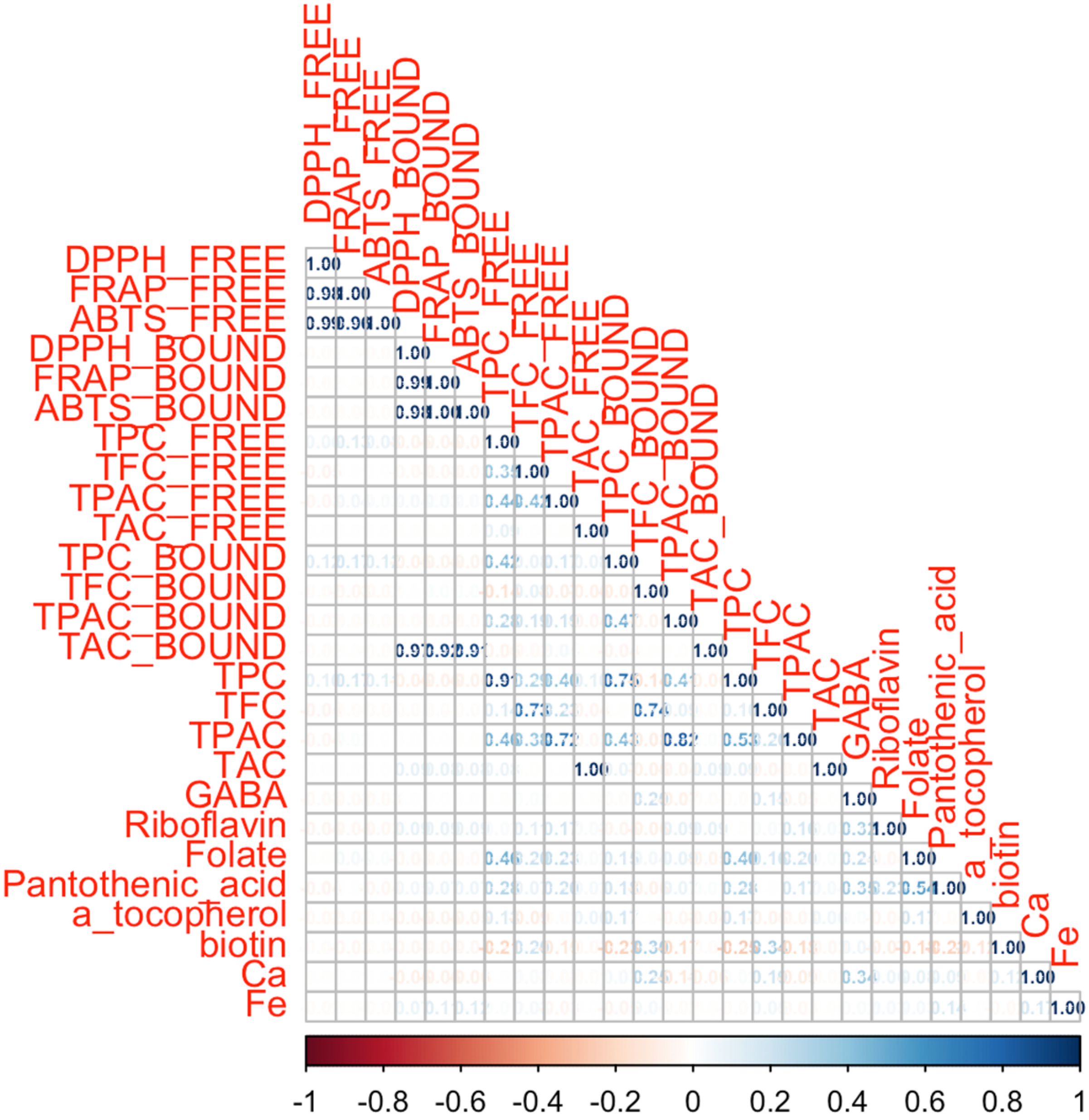
DPPH_FREE
FRAP_FREE
ABTS_FREE
DPPH_BOUND
FRAP_BOUND
ABTS_BOUND
TPC_FREE
TFC_FREE
TPAC_FREE
TAC_FREE
TPC_BOUND
TFC_BOUND
TPAC_BOUND
TAC_BOUND
TPC
TFC
TPAC
TAC
GABA
Riboflavin
Folate
Pantothenic_acid
a_tocopherol
biotin
Ca
Fe
-1
-0.8
-0.6
-0.4
-0.2
0
0.2
0.4
0.6
0.8
1

Supplementary Figure 3. Correlation plot of the traits used for the models. Only one of the highly (≥ 0.7) correlated variables was used to the model to avoid overfitting.

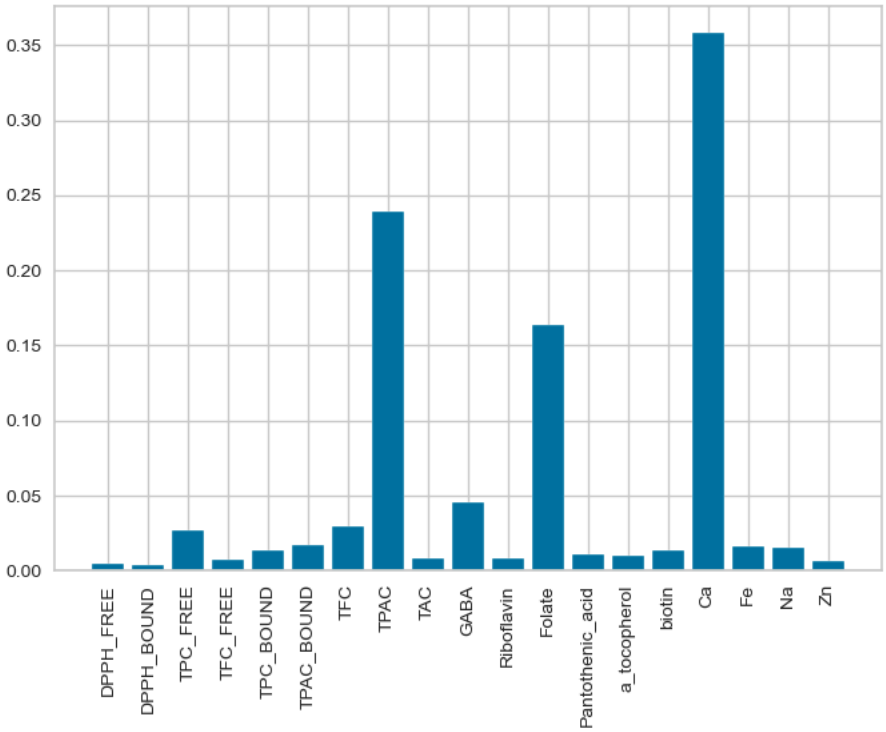

Supplementary Figure 4. The variables and their mean decrease accuracy in the random forest model

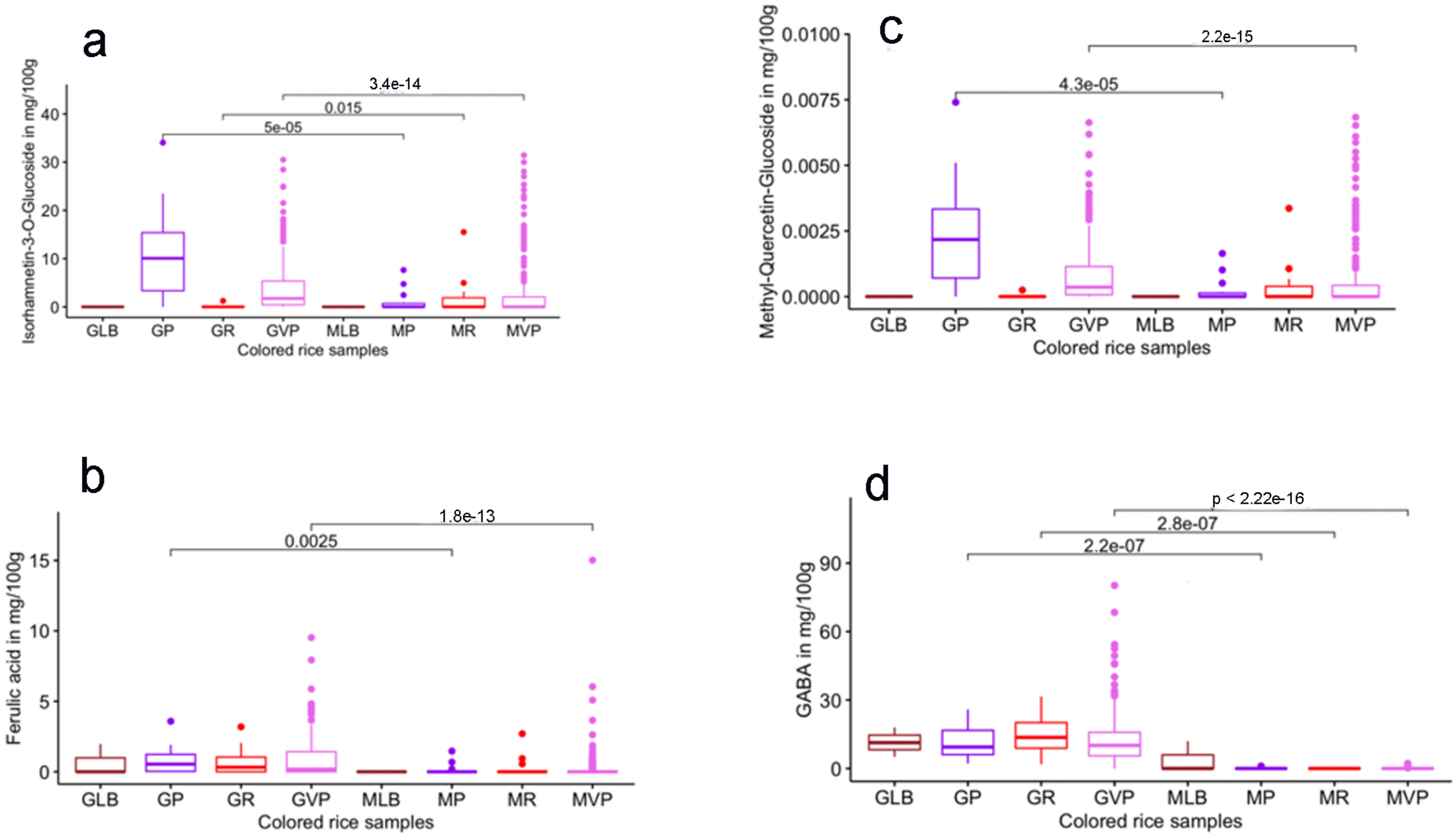

a
Isorhamnetin-3-O-Glucoside in mg/100g
3.4e-14
0.015
5e-05
40
30
20
10
0
GLB GP GR GVP MLB MP MR MVP
Colored rice samples
c
Methyl-Quercetin-Glucoside in mg/100g
0.0100
0.0075
0.0050
0.0025
0.0000
2.2e-15
4.3e-05
GLB GP GR GVP MLB MP MR MVP
Colored rice samples
b
Ferulic acid in mg/100g
1.8e-13
0.0025
15
10
5
0
GLB GP GR GVP MLB MP MR MVP
Colored rice samples
d
GABA in mg/100g
p < 2.22e-16
2.8e-07
2.2e-07
90
60
30
0
GLB GP GR GVP MLB MP MR MVP
Colored rice samples

Supplementary Figure 5. Germination has shown to increase phenolic acid, GABA, and specific glycosylated flavonoids as shown the absolute quantification reported of these compounds (units are in mg/100g). (a) Variable purple and purple showed significant increase in isorhamnetin-3-O-glucoside whereas decrease in red rice samples after the germination process, (b) Variable purple and purple showed significant increase in methyl-Quercetin Glucoside whereas decrease in red rice samples after the germination process, (c) Variable purple and purple showed significant increase in ferulic acid after the germination process, (d) Germination showed significant increase in GABA after the germination process. (GLB = Germinated Light brown, GP = Germinated Purple, GR = Germinated Red, GVP = Germinated Variable Purple, MLB = Matured (non-germinated) Light brown, MP = non-germinated Purple, MR = non-germinated Red, MVP = non-germinated variable purple). In the boxplot, the solid middle line depicts the median, while the lower and upper whiskers signify the 25th and 75th percentiles, respectively.

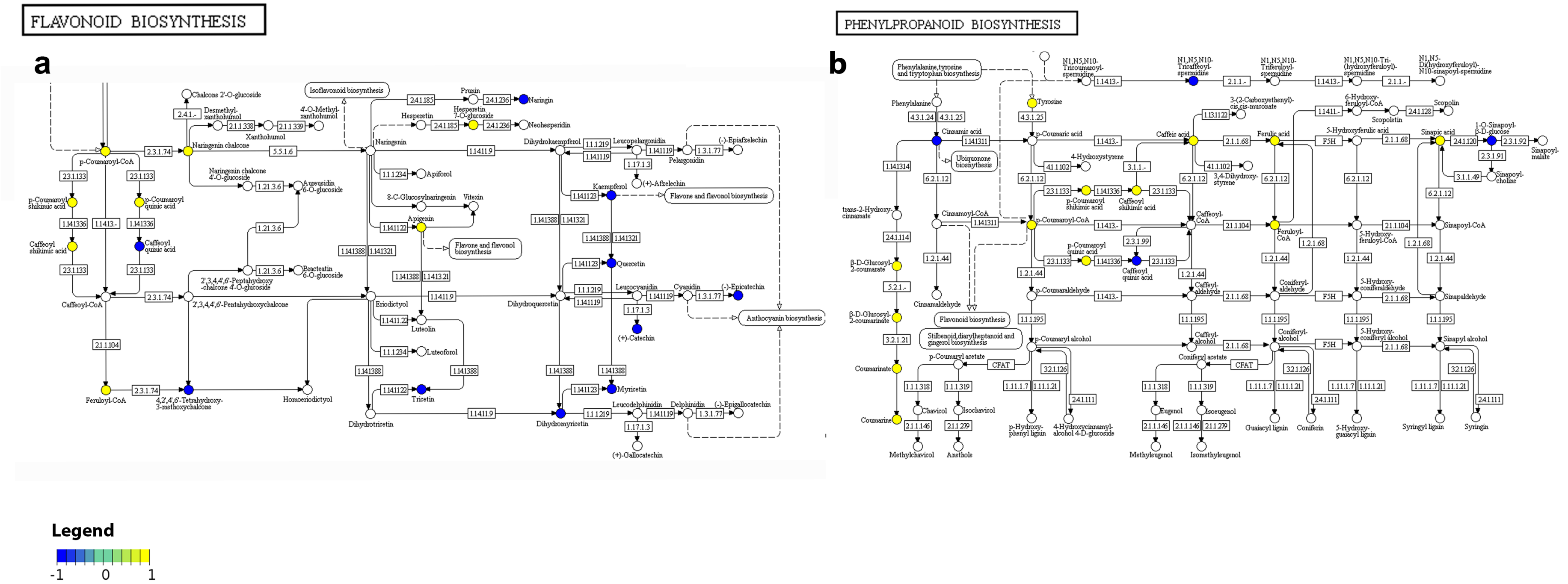

Supplementary Figure 6. Germination altered the metabolites as visualized by KEGG-enrichment analysis using *O.sativa* japonica genome. (a) Flavonoid biosynthesis has been altered upon germination favoring apigenin and coumaroyl-related flavonoids. (b) Phenylpropanoid pathway which demonstrates that phenolic acids such as caffeic acid and ferulic acids are upregulated whereas cinnamic acid is downregulated upon

germination. The number above represents the enzymes participating in the reaction. Selected enzymes that were associated with differentially accumulated metabolites: 2.3.1.133 - shikimate O-hydroxycinnamoyltransferase, 1.14.14.96 - 5-O-(4-coumaroyl)-D-quinate 3'-monooxygenase, 2.1.1.104 - caffeoyl-CoA O-methyltransferase, 2.3.1.74 - chalcone synthase, 1.14.20.6 - flavonol synthase, 1.14.14.81 - flavonoid 3',5'-hydroxylase, 1.14.14.82 - flavonoid 3'-monooxygenase, 1.2.1.44 - cinnamoyl-CoA reductase, and 1.14.14.91 - trans-cinnamate 4-monooxygenase. The metabolite peak intensities were normalized on a scale of -1 to +1, where -1 represents downregulation and +1 represents upregulation upon germination.

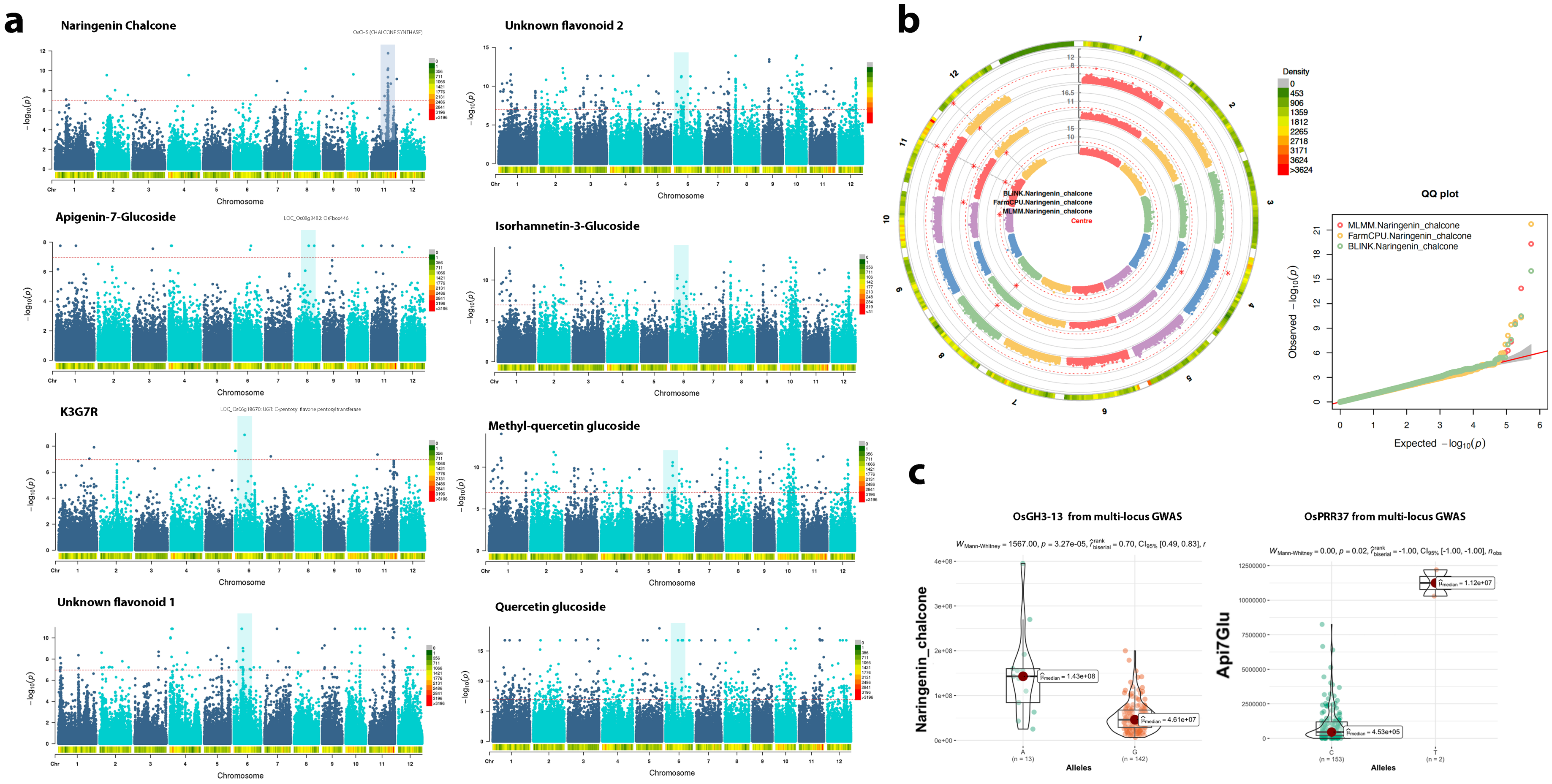

Supplementary Figure 7. Genetic analysis was used to determine genes responsible for the enrichment of specific metabolites. (a) Single-locus GWAS results of selected metabolites highlighting genetic regions associated with the flavonoid biosynthesis where key genes such as UGT and CHS are identified (b) Multi-locus GWAS of Naringenin Chalcone using FarmCPU, MLMM, and BLINK models which revealed

additional genes such as OsGH3-13 (LOC_Os11g32520) and OsDjC75 (LOC_Os11g36520), which both play a role in plant growth and development and stress tolerance. In the QQplots, MLMM represents red graph, FarmCPU represents yellow graph, and BLINK represents green graph. The gray diagonal line represents the quantiles of the theoretical distribution (e.g., normal distribution) expected if the sample perfectly follows that distribution. (c) Significant difference between the allele variants of the lead snps from OsPRR37 and OsGH3-13 genes produced in the multi-locus GWAS ($p < 0.05$). In the boxplot, the solid middle line depicts the median, while the lower and upper whiskers signify the 25th and 75th percentiles, respectively.

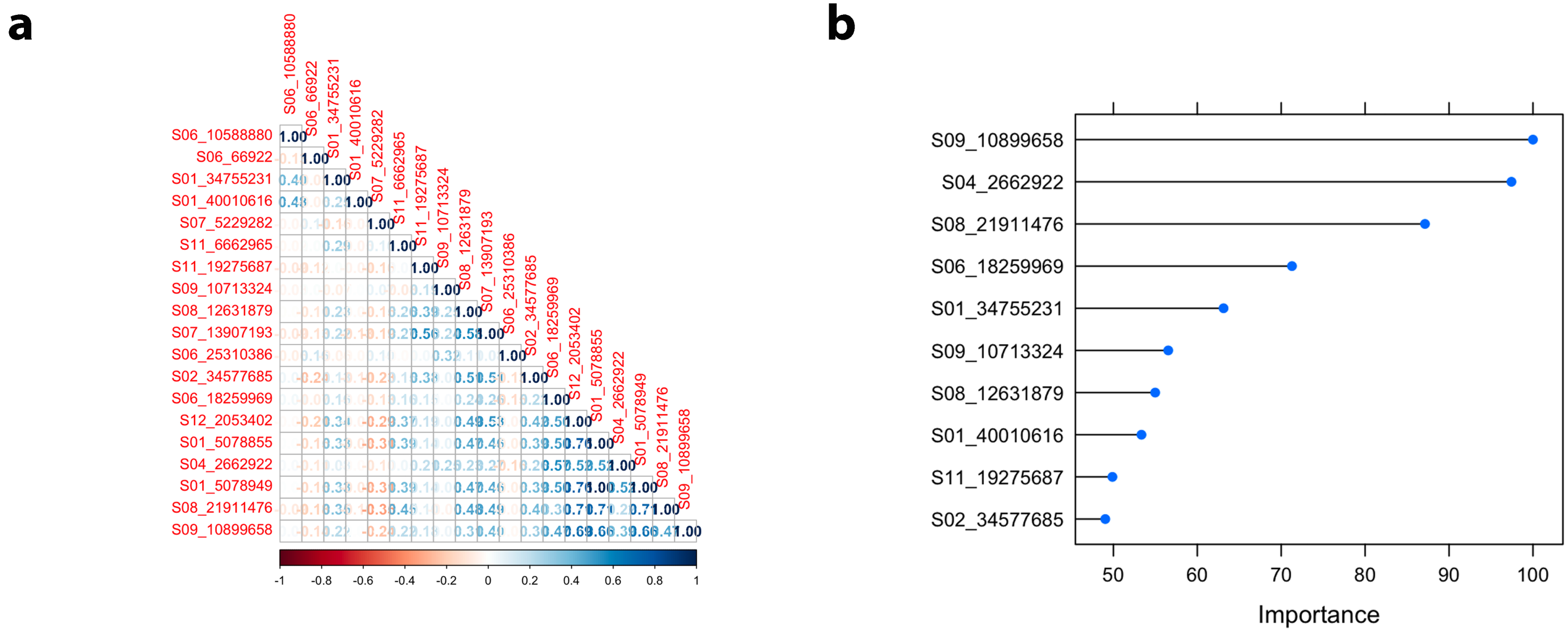

Supplementary Figure 8. Genetic markers utilized in the RF model (a) Correlation plot of the genetic markers (correlation of ≥ 0.7 were filtered to avoid overfitting) (b) Importance scores of the genetic markers used in the RF model
