# Supplementary material for: Metabolomics and machine learning technique revealed that germination enhances the multi-nutritional properties of pigmented rice
Source: Commun Biol. 2023 Oct 2;6:1000. doi: 10.1038/s42003-023-05379-9 (PMC10545681; doi:10.1038/s42003-023-05379-9)
Supplement: Supplementary file 3 — Description of Additional Supplementary Files [file 42003_2023_5379_MOESM3_ESM.pdf]

Description of Additional Supplementary Files

File name: Supplementary Data 1
Description: Mineral content of Germinated (GBCR) and non-germinated rice (MBCR).

File name: Supplementary Data 2
Description: Optimization of parameters on the Random Forest (FR) model used to classify PRS based on nutritional parameters.

File name: Supplementary Data 3
Description: Correlation of the metabolites.

File name: Supplementary Data 4
Description: Identity of the samples.

File name: Supplementary Data 5
Description: Summary GWAS result for single-locus GWAS.

File name: Supplementary Data 6
Description: Haplotype mining for UGT and CHS genes.

File name: Supplementary Data 7
Description: Metabolite reporting checklist for LC-MS.

File name: Supplementary Data 8
Description: Linear equations for standards by UPLC-Extractive Orbitrap mass spectrometer.

File name: Supplementary Data 9
Description: LC metabolite annotation.

File name: Supplementary Data 10
Description: Metabolite peak intensities of germinated and non-germinated rice.

File name: Supplementary Data 11
Description: Clustering of samples using AGNES-Ward technique.

File name: Supplementary Data 12
Description: Clustering of samples summary statistics.
